# Impact of rumination on sleep quality among patients with non‑alcoholic fatty liver disease: a moderated mediation model of anxiety symptoms and resilience

**DOI:** 10.1186/s12888-023-04572-8

**Published:** 2023-02-02

**Authors:** Xiaolin Chang, Chenxi Guo, Heng Zhou, Li Liu

**Affiliations:** 1 Outpatient Service By Famous Specialists, The First Affiliated Hospital of China Medical University, Shenyang, China; 2 Department of Clinical Nutrition, The First Affiliated Hospital of China Medical University, Shenyang, China; 3 Department of Surgical Oncology and General Surgery, The First Affiliated Hospital of China Medical University, Key Laboratory of Precision Diagnosis and Treatment of Gastrointestinal Tumors (China Medical University), Ministry of Education, Shenyang, China; 4 Department of Anesthesiology, The First Affiliated Hospital of China Medical University, No. 155 Nanjing Bei Street, Heping District, Shenyang, 110001 Liaoning China; 5 Department of Social Medicine, School of Health Management, China Medical University, No. 77 Puhe Road, Shenyang North New Area, Shenyang, 110122 Liaoning China

**Keywords:** Sleep quality, Rumination, Anxiety, Resilience, Moderated mediation, Non‑alcoholic fatty liver disease

## Abstract

**Background:**

Poor sleep raises the risk of non-alcoholic fatty liver disease (NAFLD) and hastens disease progression. It is critical to figure out what factors impact the sleep quality of NAFLD patients. The present study aimed to investigate the role of anxiety symptoms in accounting for the impact of rumination on sleep quality and the moderating role of resilience on the associations of rumination with anxiety symptoms and sleep quality.

**Methods:**

In the cross-sectional study, 285 NAFLD patients completed the Chinese version of the Pittsburgh Sleep Quality Index, the Ruminative Responses Scale, the Generalized Anxiety Disorder 7-item scale, and the 14-item Resilience Scale to measure sleep quality, rumination (including brooding and reflection), anxiety symptoms, and resilience, respectively. The PROCESS macro for SPSS v4.0 procedure was applied to perform moderated mediation analysis.

**Results:**

The roles of anxiety symptoms in accounting for the positive associations of brooding, reflection and rumination with poor sleep quality were revealed. It was found that there was a significant moderating role of resilience on the positive associations of brooding, reflection and rumination with anxiety symptoms, which were gradually reduced as resilience increased. The direct associations between brooding, reflection and rumination and poor sleep quality were not significantly moderated by resilience. Thus, a moderated mediation model involving anxiety symptoms and resilience for explaining the impact of rumination on poor sleep quality was supported among patients with NAFLD.

**Conclusions:**

Rumination (including brooding and reflection) could be positively related to poor sleep quality, and anxiety symptoms had a significant role in accounting for the relationship among patients with NAFLD. Resilience showed a moderating role that could attenuate the positive association between rumination and anxiety symptoms. Interventions aimed at alleviating rumination, reducing anxiety symptoms, and enhancing resilience could improve the sleep quality of NAFLD patients.

**Supplementary Information:**

The online version contains supplementary material available at 10.1186/s12888-023-04572-8.

## Background

Non-alcoholic fatty liver disease (NAFLD) is a condition in which the liver accumulates excessive fat in the absence of a minor cause such as alcohol or drugs. NAFLD encompasses the entire course of this disease, including non-alcoholic fatty liver, non-alcoholic steatohepatitis (NASH), fibrosis, and cirrhosis [1]. Over the last 30 years, NAFLD has been prevalent, affecting more than 25 percent of the world’s population, with the highest prevalence rate of 35.7% and 35.3% in South America and North America, respectively [2]. According to a recent systematic review and meta-analysis, the overall prevalence of NAFLD is 29.2% in China [3]. Despite the regional difference, the prevalence of NAFLD is increasing overall worldwide [2, 3]. There is evidence that NAFLD significantly increases overall mortality, and the mortality rate of NAFLD patients is higher than that of the general population [4, 5]. NAFLD places an increasing burden on patients and their families and poses a major public health challenge [6, 7].

NAFLD is a metabolic disorder with an unhealthy lifestyle [8], such as sleep insufficiency and poor sleep quality [9]. Epidemiological research has shown that sleep insufficiency and poor sleep increase the risk of NAFLD [9–11]. Three biological mechanisms have been demonstrated, including poor sleep (1) may reduce the sensitivity of insulin and raise the incidence of type-2 diabetes [12, 13]; (2) can trigger inflammatory reactions (e.g., an increase in interleukin-6 and tumor necrosis factor-α) [14, 15]; (3) can activate the hypothalamic–pituitary–adrenal axis to secrete stress hormones (e.g., catecholamine, cortisol), raising the risk of metabolic syndrome [16, 17]. Moreover, poor sleep can aggravate NAFLD’s progression [18]. Therefore, identifying risk and protective factors of sleep quality and clarifying their mechanism of action may help improve health outcomes among patients with NAFLD.

Rumination refers to repeated passive thinking, including obsessive thinking and introspective meditation, in which individuals think frequently about adverse life events, including suffering from an illness [19]. Harvey’s cognitive model of insomnia indicates that the arousal and distress caused by excessive negative cognition are crucial in causing and maintaining sleep problems [20]. The cognitive approach in this model provides a framework for understanding and treating sleep problems. The cognitive model suggests that rumination and worries about stressful life events disrupt one’s sleep. Numerous studies have demonstrated a positive relationship between rumination and poor sleep [21, 22]. NAFLD could be considered as such an adverse life event that can induce patients’ repeated passive thinking. Hence, reducing rumination could help cope with the sleep disturbances associated with NAFLD. In addition, as two sub-factors of rumination, brooding and reflection have been proposed by Treynor et al. in a psychometric analysis of rumination [23]. Brooding is a tendency to cling to negative situations or actions, while reflection refers to considering the reasons for these negative situations or actions. It is important to distinguish these two components of rumination and measure them separately because they have different relationships with emotional outcomes [23].

Although the initial concept of rumination focused on depression and its causes and effects, more and more studies have expanded the concept of rumination, including the attention to adverse life events experienced by individuals. Increasing evidences have demonstrated that rumination may also be associated with other negative emotional outcomes, such as anxiety [24, 25] and post-traumatic stress disorder [26, 27], following various adverse life events. Therefore, the impact of rumination on sleep quality might be accounted for by other emotional variables. Anxiety symptoms are common among patients with NAFLD, and multiple studies have suggested a potential association between NAFLD and anxiety disorder [28, 29]. Anxiety symptoms refer to a group of negative mood states that is characterized by excessive worry that interferes with a person’s ability to function normally. The psychological process involved in coping with adverse life events (e.g., NAFLD) starts from the cognitive process (e.g., rumination) and then enters into the emotion and affection process (e.g., anxiety symptoms). Thus, we postulated that anxiety symptoms could be a psychological outcome of rumination in this study.

According to previous studies [30, 31], anxiety could be a predictor of sleep insufficiency and poor sleep quality among NAFLD patients. Anxiety and sleep are intricately connected through a self-reinforcing feedback loop [32]. Anxiety symptoms can prevent individuals from falling asleep or staying asleep, and make it difficult to get restful sleep regularly. Therefore, anxiety symptoms could account for the impact of rumination on poor sleep quality among NAFLD patients. At present, whether rumination and anxiety symptoms act on the sleep quality of patients with NAFLD is uncertain, and the relevant mechanism also needs to be clarified.

Resilience is an ability that allows individuals to adjust successfully to challenging situations [33]. Resilience can alter a person’s risk assessment, cognition in reaction to sensed emotions, and coping strategy selection. It has been discovered to have a protective effect on preventing physical and mental health issues [34]. It may help patients with NAFLD reduce the potentially harmful effects of rumination. Research has also found that individuals may mitigate miserable experiences (e.g., anxiety, post-traumatic stress disorder) via resilience [35, 36]. Kumpfer’s theory of resilience framework states that positive resilience may help individuals resist negative emotions in their lives, thus achieving a higher level of resilience, which is a process of positive feedback [34]. Therefore, it can be inferred that resilience could counteract the impact of rumination on sleep quality in patients with NAFLD [21, 35]. In addition, rumination refers to a cognitive process focused on adverse life events, whereas both anxiety symptoms and sleep quality could be considered the negative emotional and behavioral outcomes of the passive cognitive process. In the psychological process of coping with NAFLD, resilience, as a positive psychological capability, would play a moderating role on the relationship between cognitive style and emotional and behavioral outcomes. However, to date, no research has discussed the resilience’s moderating role on the relationships of rumination with anxiety symptoms and sleep quality among NAFLD patients.

**Fig. 1:**
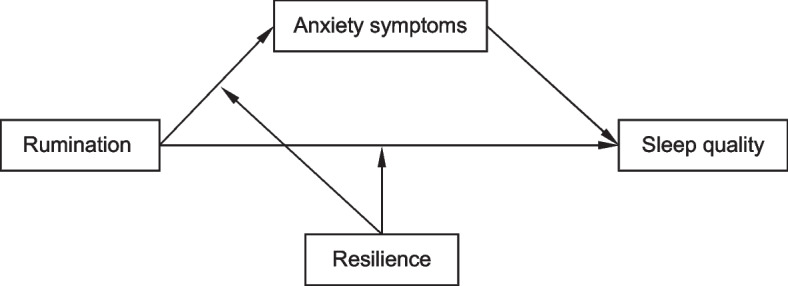
Hypothetical model

Given the theory and evidence concerned above, this present study aimed to verify these hypotheses among patients with NAFLD: (1) rumination (including brooding and reflection) has a positive relationship with poor sleep quality and anxiety symptoms; (2) anxiety symptoms would account for the link between rumination and sleep quality; (3) resilience plays a moderating role on the associations of rumination with anxiety symptoms and sleep quality. Specifically, we put anxiety symptoms as the mediator and resilience as the moderator in an integrated model explaining the mechanism of rumination on sleep quality (Fig. 1), which references Hayes’s study [37]. This research will help us better understand the relationship between rumination and sleep quality, and the roles of anxiety symptoms and resilience in the relationship among patients with NAFLD. In clinical practice, the results of this study will suggest potential intervention targets for coping with poor sleep quality in this population. In future research and practice, targeted intervention will be implemented to improve sleep quality and manage NAFLD well.

## Methods

### Study design and sample

In the cross-sectional survey, consecutive outpatients diagnosed with NAFLD were recruited at the Department of Digestive Diseases of the First Affiliated Hospital of China Medical University from September 1, 2021, to January 31, 2022, which is a leading provider of digestive disease treatment in Liaoning Province, Northeast China. Participants were recruited if they (1) were 18 years or older, (2) had been diagnosed with NAFLD by ultrasonography, computed tomography, or magnetic resonance imaging during the previous 24 months or by biopsies within the previous 36 months, (3) were able to communicate in Chinese well to complete a questionnaire with clear consciousness and cognition. Participants were excluded if they had (1) liver injury caused by other reasons (such as hepatitis virus or excessive drinking), steatosis caused by other reasons (such as glucocorticoid or tamoxifen), decompensated liver disease or hepatocellular carcinoma, (2) a history of serious psychiatric illness, and (3) cognitive impairment. All eligible patients were recruited to participate in our study, and they were asked to complete a set of self-administered questionnaires. Clinical data were obtained from each participant’s medical record. The China Medical University’s Ethics Committee approved to the study. The research procedures follow the Declaration of Helsinki’s principles. All participants had signed written informed consent for their participation and to access their medical records.

In total, 300 eligible patients agreed to participate and complete questionnaires. Any data missing on item level regarding the questionnaires were excluded from analyses, including 15 responses (3 responses for sleep quality, 1 response for rumination, 2 responses for resilience, and 9 responses for clinical characteristics). Thus, effective responses were obtained from 285 (95.0%) participants.

### Measurement of sleep quality

Patients’ sleep quality during the previous month was measured using the Chinese version of the Pittsburgh Sleep Quality Index (PSQI) [38, 39]. The assessment includes 18 self-reported items. Subjective sleep quality, sleep latency, sleep length, sleep efficiency, sleep disruptions, use of sleep medicine, and daytime dysfunction are the seven categories of sleep that are integrated [38]. The first four items of the PSQI are in a free-response format and used to assess sleep latency, duration of sleep, and sleep efficiency, respectively. For the remaining 14 items with a 4-point Likert scale, participants indicate the frequency of causes of trouble sleeping, taking medicine, and having trouble staying awake (from 0 = not during the past month to 3 = three or more times a week), overall sleep quality (from 0 = very good to 3 = very bad), and keeping up enthusiasm (from 0 = no problem at all to 3 = a very big problem). The score range of each sleep’s category is 0 to 3. The seven category scores are summed to get a total score of sleep quality (0 to 21). Higher values indicate worse sleep quality. The PSQI had a Cronbach’s α coefficient of 0.74 in this sample.

### Measurement of rumination

The Chinese version of the Nolen-Hoeksema Ruminative Responses Scale (RRS) [40, 41] was adopted to assess rumination. To measure a general concept of rumination, the brooding (5 items) and reflection (5 items) subscales of the RRS were used in the present study. The brooding and reflection scores and the total score of rumination (the sum of brooding and reflection items) was used to predict sleep quality in the subsequent analyses. On a 4-point Likert scale, participants indicate how often they engage in a ruminative thinking style when they feel sick (NAFLD) from 1 = almost never to 4 = always. A brooding or reflection score may vary between 5 and 20, while a total rumination score may vary between 10 and 40, with higher values suggesting greater rumination. Cronbach’s α coefficients for the brooding and reflection subscales and the whole RRS were 0.84, 0.79, and 0.89 in our sample.

### Measurement of anxiety symptoms

The Chinese version of the Generalized Anxiety Disorder 7-item (GAD-7) scale was used to measure anxiety symptoms in the previous two weeks [42, 43]. The GAD-7 lists seven key symptoms of generalized anxiety, and participants rate how often they experience these symptoms on a 4-point Likert scale ranging from 0 = not at all to 3 = almost every day. The seven item scores are summed to get a total score of anxiety symptoms ranging from 0 to 21. Higher values reflect severer anxiety symptoms. In our sample, the GAD-7 scale had a Cronbach’s α coefficient of 0.91.

### Measurement of resilience

The Chinese version of the 14-item Resilience Scale (RS-14) was adopted to measure resilience [44, 45]. The RS-14 is a single-factor structure instrument. Participants express their levels of agreement for each item on a 7-point Likert scale, from 1 = strongly disagree to 7 = strongly agree. The 14 item scores are summed to get a total score of resilience, which may vary between 14 and 98, with higher values indicating more resilience. In this sample, the RS-14’s Cronbach’s α coefficient was 0.95.

### Demographic characteristics

Demographic characteristics of patients included age (years), gender, marital status, educational level, place of residence, occupation, and monthly household income (Chinese Yuan, CNY). Age was collected as a continuous variable and divided into three groups: 18–39, 40–59, and ≥ 60 years. Marital status was categorized as married/cohabited and single/divorced/widowed/separated. Educational level was classified as junior high school or below, senior high school, junior college, and college or above. Place of residence was categorized as urban and rural. Occupation was categorized as yes or no. Monthly household income was sorted into three groups: < 5,000, 5,000–10,000, and > 10,000 CNY.

### Clinical characteristics

Body mass index (BMI, kg/m^2^), duration of suffering from NAFLD (months), disease severity, comorbidity, treatment, and laboratory examination were gathered as clinical characteristics. BMI was collected as a continuous variable and divided into three groups: < 24, 24–28, and ≥ 28 kg/m^2^. The duration of suffering from NAFLD was classified as ≤ 6 and > 6 months. NAFLD is a continuous spectrum of liver diseases. Simple fatty liver can progress to NASH, and then to fibrosis and cirrhosis. As the disease progresses, the severity of NAFLD increases. Comorbidity and treatment were categorized as yes or no. Laboratory examination included alanine aminotransferase (ALT, U/L), aspartate aminotransferase (AST, U/L), and albumin (g/L).

### Statistical analysis

Descriptive statistics were adopted to analyze demographic and clinical variables. T-tests and one-way ANOVA were performed to determine group differences. The correlation was tested using Pearson’s correlation analysis. The Model 4 and Model 8 of the PROCESS macro for SPSS v4.0 procedure were used to examine the mediation and moderated mediation [37]. The PSQI score was modeled as the dependent variable, while brooding, reflection and rumination were treated as the independent variables separately. For explaining the impact of rumination on sleep quality, anxiety symptoms served as the mediator and resilience as the moderator. In the analyses, we standardized the six variables to remove scale differences. The bootstrapping sample size was set to 5,000, and a 95% confidence interval (95% CI) did not include 0, indicating a statistically significant mediation. Simple slope analysis (pick-a-point method) was carried out to demonstrate the moderating role of resilience. Based on the mean and standard deviation (SD) of resilience, it was split into low (1 SD below the mean), medium (the mean), and high (1 SD above the mean). The SPSS 21.0 for Windows (IBM, Asia Analytics Shanghai) was used for statistical analysis and a two-tailed *P* < 0.05 was regarded as statistically significant.

## Results

### Demographic and clinical characteristics of subjects

Table 1 shows the demographic and clinical characteristics of subjects. On average, the patients were 43.2 years old (SD = 13.4). Of these subjects, 147 (51.6%) were male, 84.2% (240) were married/cohabited, 162 (56.9%) had an educational level of junior college or above, 245 (86.0%) lived in urban areas, 222 (77.9%) had an occupation, and 119 (41.8%) reported a monthly household income of less than 5,000 CNY. For clinical characteristics, the mean BMI was 27.3 kg/m^2^ (SD = 3.7). Among these subjects, 196 (68.8%) had a duration of NAFLD ≤ 6 months, the majority of subjects (243, 85.3%) were simple fatty liver, 153 (53.7%) had comorbidities, and 117 (41.1%) underwent a treatment.

**Table 1:** Demographic and clinical characteristics of subjects

Variables	Patients (*n* = 285)
Demographic characteristics	
Age (years), Mean ± SD	43.2 ± 13.4
Age group (*n*, %)	
18–39 years	122 (42.8)
40–59 years	135 (47.4)
≥ 60 years	28 (9.8)
Gender (*n*, %)	
Male	147 (51.6)
Female	138 (48.4)
Marital status (*n*, %)	
Married/cohabited	240 (84.2)
Single/divorced/widowed/separated	45 (15.8)
Educational level (*n*, %)	
Junior high school or below	58 (20.4)
Senior high school	65 (22.8)
Junior college	66 (23.2)
College or above	96 (33.7)
Place of residence (*n*, %)	
Urban	245 (86.0)
Rural	40 (14.0)
Occupation (*n*, %)	
Yes	222 (77.9)
No	63 (22.1)
Monthly household income (*n*, %)	
< 5,000 CNY	119 (41.8)
5,000–10,000 CNY	116 (40.7)
> 10,000 CNY	50 (17.5)
Clinical characteristics	
BMI (kg/m^2^), Mean ± SD	27.3 ± 3.7
BMI group (*n*, %)	
< 24 kg/m^2^	48 (16.8)
24–28 kg/m^2^	137 (48.1)
≥ 28 kg/m^2^	100 (35.1)
Duration of NAFLD (*n*, %)	
≤ 6 months	196 (68.8)
> 6 months	89 (31.2)
Disease severity (*n*, %)	
Simple fatty liver	243 (85.3)
NASH	7 (2.5)
Fibrosis	10 (3.5)
Cirrhosis	3 (1.1)
Unknown	22 (7.7)
Comorbidities (*n*, %)	
Yes	153 (53.7)
Hyperlipidemia	30
Diabetes	26
Hypertension	36
Cardiovascular diseases	13
Others	48
No	132 (46.3)
Treatment (*n*, %)	
Yes	117 (41.1)
No	168 (58.9)
Laboratory examination, Median (IQR)	
ALT (U/L)	52.0 (23.0, 81.0)
AST (U/L)	36.0 (21.0, 48.9)
Albumin (g/L)	44.9 (42.7, 47.1)

*ALT* Alanine aminotransferase, *AST* Aspartate aminotransferase, *BMI* Body mass index, *CNY* Chinese Yuan, *IQR* Interquartile range, *NAFLD* Non-alcoholic fatty liver disease, *NASH* Non-alcoholic steatohepatitis, *SD* Standard deviation

### Correlations among rumination, anxiety symptoms, resilience, and PSQI score

Descriptive statistics for rumination, anxiety symptoms, resilience and PSQI score, and correlations among them are displayed in Table 2. The PSQI score revealed a strong positive correlation with anxiety symptoms (*r* = 0.524, *P* < 0.01) and a weak positive correlation with brooding (*r* = 0.252, *P* < 0.01), reflection (*r* = 0.272, *P* < 0.01), and rumination (*r* = 0.280, *P* < 0.01). However, it was negatively and weakly correlated with resilience (*r* = -0.230, *P* < 0.01). Anxiety symptoms showed a moderate positive correlation with brooding (*r* = 0.405, *P* < 0.01), reflection (*r* = 0.388, *P* < 0.01), and rumination (*r* = 0.426, *P* < 0.01) and a moderate negative correlation with resilience (*r* = -0.396, *P* < 0.01). There were strong positive correlations among brooding, reflection and rumination. Resilience was inversely and weakly correlated with brooding (*r* = -0.239, *P* < 0.01), reflection (*r* = -0.233, *P* < 0.01), and rumination (*r* = -0.253, *P* < 0.01).

**Table 2:** Correlations among rumination, anxiety symptoms, resilience and PSQI score

Variables	Mean ± SD	1	2	3	4	5
1. PSQI	5.21 ± 3.34	1				
2. Anxiety symptoms	3.85 ± 4.30	0.524**	1			
3. Brooding	8.42 ± 3.12	0.252**	0.405**	1		
4. Reflection	7.41 ± 2.70	0.272**	0.388**	0.737**	1	
5. Rumination	15.83 ± 5.42	0.280**	0.426**	0.942**	0.921**	1
6. Resilience	76.62 ± 15.43	-0.230**	-0.396**	-0.239**	-0.233**	-0.253**

*PSQI* Pittsburgh sleep quality index, *SD* Standard deviation

^**^P < 0.01

### Mediation model

The results of mediation model are presented in Table 3. Path coefficients included: *c* (the association between brooding, reflection and rumination and PSQI score), *a* (the association between brooding, reflection and rumination and anxiety symptoms), *b* (the association between anxiety symptoms and PSQI score), *c’* (the association between brooding, reflection and rumination and PSQI score after adding anxiety symptoms), and *a* × *b* (the product of *a* and *b*, which indicates the size of mediation). In univariate analysis (Additional file 1), demographic and clinical variables including age, gender, occupation, monthly household income, duration of NAFLD, and disease severity were related to anxiety symptoms or PSQI score (*P* < 0.1). Thus, these variables were added to the regression models as covariates. Anxiety symptoms showed a significant role in accounting for the positive associations of brooding (*a* × *b* = 0.187, 95% CI: 0.110 to 0.273), reflection (*a* × *b* = 0.169, 95% CI: 0.093 to 0.255) and rumination (*a* × *b* = 0.189, 95% CI: 0.111 to 0.274) with PSQI score, respectively. The proportions (*a* × *b*/*c* × 100%) of anxiety symptoms’ roles were 77.3%, 64.3%, and 70.3%, respectively. For the brooding model, the model’s explanatory power (*R*^*2*^) for anxiety symptoms and PSQI score reached 21.7% and 30.4%, respectively. For the reflection model, the *R*^*2*^ for anxiety symptoms and PSQI score reached 19.7% and 30.9%, respectively. And for the rumination model, the *R*^*2*^ for anxiety symptoms and PSQI score reached 22.7% and 30.7%, respectively.

**Table 3:** Results of mediation model

Models	Path coefficients				*a* × *b* (95% CI)
	***c***	***a***	***b***	***c’***	
Brooding	0.242**	0.383**	0.488**	0.055	0.187 (0.110, 0.273)
Reflection	0.263**	0.355**	0.475**	0.095	0.169 (0.093, 0.255)
Rumination	0.269**	0.396**	0.477**	0.080	0.189 (0.111, 0.274)

Standardized coefficients were displayed. Age, gender, occupation, monthly household income, duration of NAFLD, and disease severity were adjusted

*CI* Confidence interval

^**^P < 0.01

### Moderated mediation model

**Fig. 2:**
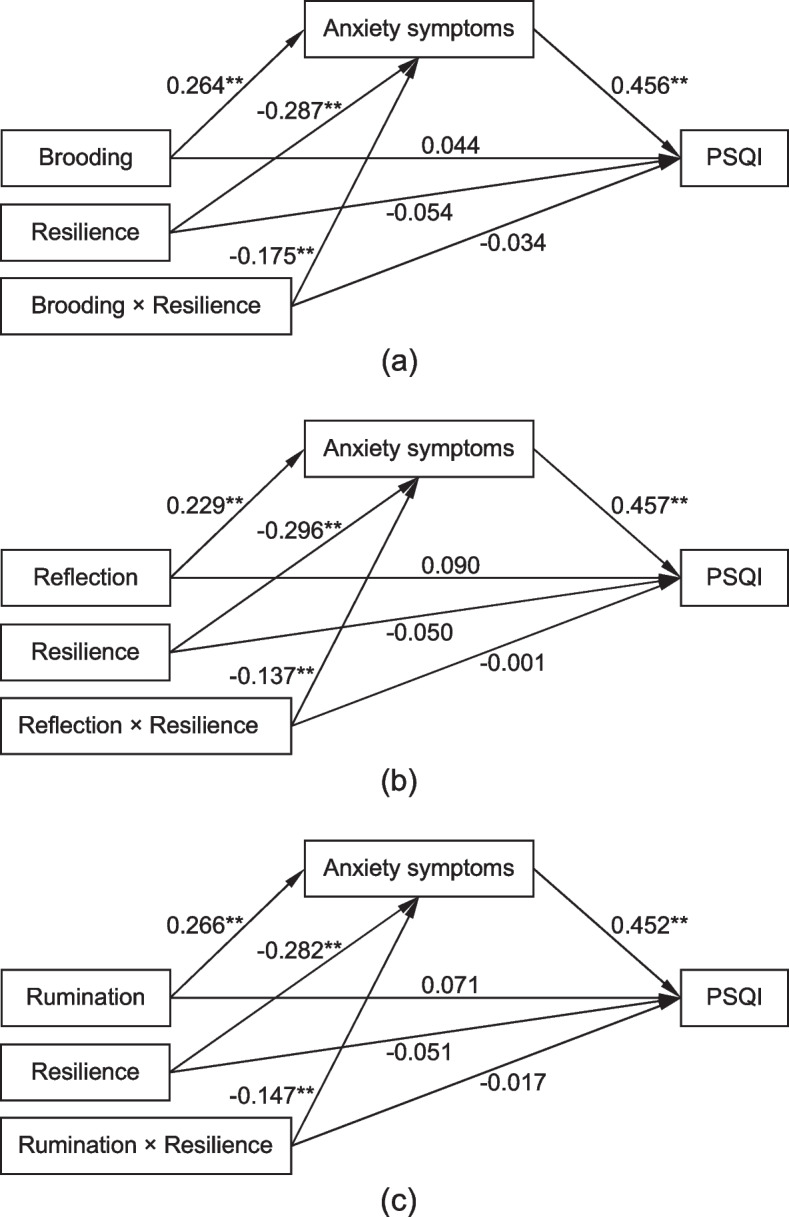
Moderated mediation model. **(a)** Moderated mediation model for brooding and PSQI; (**b**) Moderated mediation model for reflection and PSQI; (**c**) Moderated mediation model for rumination and PSQI. Standardized path coefficients were displayed. Age, gender, occupation, monthly household income, duration of NAFLD, and disease severity were adjusted. PSQI: Pittsburgh Sleep Quality Index. **P < 0.01

**Fig. 3:**
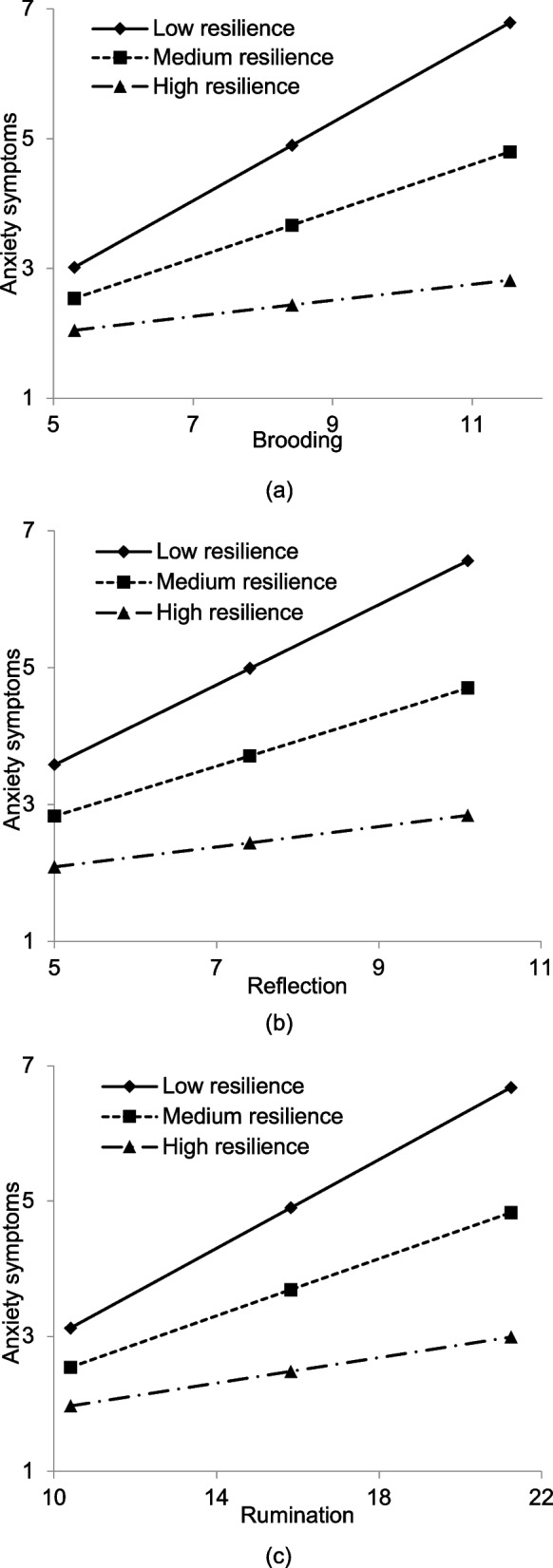
Moderating role of resilience on the association between rumination and anxiety symptoms. **(a)** Moderating role of resilience on the association between brooding and anxiety symptoms; (**b**) Moderating role of resilience on the association between reflection and anxiety symptoms; (**c**) Moderating role of resilience on the association between rumination and anxiety symptoms. Low resilience: 1 SD below the mean; Medium resilience: the mean; High resilience: 1 SD above the mean. Age, gender, occupation, monthly household income, duration of NAFLD, and disease severity were adjusted

The results of moderated mediation model are displayed in Fig. 2. The associations of brooding (*β* = -0.175, *P* < 0.01), reflection (*β* = -0.137, *P* < 0.01) and rumination (*β* = -0.147, *P* < 0.01) with anxiety symptoms was significantly moderated by resilience. An extra 4.9% 3.3%, and 3.9% of the variance in anxiety symptoms was explained by the interactions of brooding, reflection and rumination with resilience. These associations were gradually reduced as resilience increased: for the brooding-anxiety symptoms association, low resilience (*β* = 0.438, *P* < 0.001), medium resilience (*β* = 0.264, *P* < 0.001), high resilience (*β* = 0.089, *P* = 0.210); for the reflection-anxiety symptoms association, low resilience (*β* = 0.366, *P* < 0.001), medium resilience (*β* = 0.229, *P* < 0.001), high resilience (*β* = 0.092, *P* = 0.219); and for the rumination-anxiety symptoms association, low resilience (*β* = 0.414, *P* < 0.001) medium resilience (*β* = 0.266, *P* < 0.001), high resilience (*β* = 0.119, *P* = 0.100). The moderating roles of resilience are plotted in Fig. 3. The association between brooding, reflection and rumination and PSQI score was not significantly moderated by resilience. For the brooding model, the *R*^*2*^ for anxiety symptoms and PSQI score reached 34.9% and 30.8%, respectively. For the reflection model, the *R*^*2*^ for anxiety symptoms and PSQI score reached 32.2% and 31.1%, respectively. And for the rumination model, the *R*^*2*^ for anxiety symptoms and PSQI score reached 34.7% and 31.0%, respectively.

As shown in Table 4, with increased resilience, the role of anxiety symptoms in accounting for the association between brooding and PSQI score was steadily reduced (low resilience: 0.200, 95% CI: 0.118, 0.302; medium resilience: 0.120, 95% CI: 0.064, 0.192; high resilience: 0.041, 95% CI: -0.018, 0.109). For the reflection model, the role of anxiety symptoms was: low resilience (0.167, 95% CI: 0.098, 0.265), medium resilience (0.105, 95% CI: 0.051, 0.176), high resilience (0.042, 95% CI: -0.027, 0.118). And for the rumination model, the role of anxiety symptoms was: low resilience (0.187, 95% CI: 0.115, 0.289), medium resilience (0.121, 95% CI: 0.064, 0.192) high resilience (0.054, 95% CI: -0.011, 0.126).

**Table 4:** Results of moderated mediation model

Models	Resilience	Role of anxiety symptoms	95% CI
Brooding	Low^a, b^	0.200	0.118, 0.302
	Medium^a^	0.120	0.064, 0.192
	High	0.041	-0.018, 0.109
Reflection	Low^a, b^	0.167	0.098, 0.265
	Medium^a^	0.105	0.051, 0.176
	High	0.042	-0.027, 0.118
Rumination	Low^a, b^	0.187	0.115, 0.289
	Medium^a^	0.121	0.064, 0.192
	High	0.054	-0.011, 0.126

Low resilience, 1 SD below the mean; Medium resilience, the mean; High resilience, 1 SD above the mean. Standardized coefficients were displayed. Age, gender, occupation, monthly household income, duration of NAFLD, and disease severity were adjusted

*CI* Confidence interval

^a^compared with “High resilience”, *P* < 0.05

^b^compared with “Medium resilience”, *P* < 0.05

The model’s explanatory power for anxiety symptoms and PSQI score ranged from 19.7% to 34.9%. A set of power analyses were determined using a post-hoc method based on achieved *R*^*2*^ (0.197 to 0.349 based on different models), sample size (285), *α* (0.05), and the number of predictors (9 or 10 in different models). As a result, the present study had a statistical power level more than 0.999.

## Discussion

This is the first study to illustrate the roles of anxiety symptoms and resilience in the underlying mechanism of rumination (including brooding and reflection) with sleep quality in patients with NAFLD. The positive association between rumination and poor sleep quality could be accounted for by anxiety symptoms. Furthermore, the impact of rumination on anxiety symptoms could be moderated by resilience, and the positive association between rumination and anxiety symptoms was significant in NAFLD patients with low and medium resilience, but not those with high resilience. Although there was no significant moderating role of resilience on the direct effect of rumination on sleep quality, resilience could attenuate the role of anxiety symptoms in accounting for the positive association between rumination and sleep quality among patients with NAFLD.

Rumination was shown to be positively linked to poor sleep quality and anxiety symptoms in our study, which was consistent with the first hypothesis. This is in line with earlier studies, which have shown that the more ruminant a person is, the more anxious he/she is, and the opposite is true [24, 25]. As a passive cognitive process, rumination may be a mechanism that links stressful life events with adverse emotional outcomes, such as depressive and anxiety symptoms. Rumination reflects an individual’s passive and repetitive thinking about the present and past symptoms, losses, and failures. Individuals with ruminant response styles tend to regulate cognitive activity adversely, triggering autonomous arousal and emotional suffering, according to Harvey’s cognitive model of insomnia [20]. Another important finding was that there were basically consistent associations of brooding, reflection and rumination with anxiety symptoms and poor sleep quality among patients with NAFLD. The finding is contrary to previous findings suggested that brooding is cognitively maladaptive and more strongly associated with depressive and anxiety symptoms and sleep problems than reflection [23, 46–48]. Reflection is adaptive in reducing negative affect because it can prompt effective problem-solving [23]. Therefore, the finding should be interpreted with caution and needs to be further verified as different samples.

In this study, NAFLD patients with anxiety symptoms could suffer from poor sleep quality, partially because of their overthinking or intrusive worries. Anxious state can make individuals preferentially concentrate on internal and external sleep-related hazard indicators, such as NAFLD’s symptoms and unsupportive environment. Unfortunately, excessive and escalating anxiety may eventually be related to poor sleep. Our findings also revealed that anxiety symptoms could account for the relationships of brooding, reflection and rumination with poor sleep quality among the NAFLD patients. Rumination was indirectly related to poor sleep quality through anxiety symptoms. Higher brooding, reflection and rumination were associated with higher level of anxiety symptoms, which was correlated with poor sleep quality. This finding deserves attention in terms of anxiety symptoms treatment for improving sleep quality in NAFLD patients [49].

Our research also discovered the moderating role of resilience on the indirect effects of brooding, reflection and rumination on sleep quality through anxiety symptoms. To be specific, as resilience increased, the role of anxiety symptoms in accounting for the relationship between rumination and poor sleep quality reduced. Previous research on resilience’s moderating effects suggested that it has a protective role [21, 35]. High resilience weakened the impacts of perceived stress, anxiety, stressful life events, and rumination on sleep quality. In our study, resilience was negatively associated with anxiety symptoms and could attenuate the positive association between rumination and anxiety symptoms. In brief, resilience might improve sleep quality in indirect and moderate ways among patients with NAFLD. However, resilience did not show a moderating role on the direct impacts of brooding, reflection and rumination on sleep quality. Li et al. found that compared with college students with higher resilience, those with lower resilience had a stronger positive relationship between rumination and poor sleep quality [21]. As a result, further study is required to corroborate present results across a wide range of groups, including NAFLD patients with different cultural backgrounds and disease severity, as well as patients with other liver diseases, even the general population.

These new findings of this study could help clarify the impact of rumination on sleep quality among patients with NAFLD and provide insights for managing sleep-related risk and protective factors. Rumination mainly involves repetitive negative thoughts. Cognitive behavioral therapy can change negative thinking and behavioral patterns. Rumination-focused and mindfulness-based cognitive behavioral therapy has been demonstrated to be effective in reducing rumination [50]. Thus, NAFLD patients with poor sleep quality could benefit from cognitive behavioral training and mindfulness. Tousignant et al. have confirmed that sleep quality could be improved through interventions that reduce negative thinking about unfortunate events or enhance positive encouragement (e.g., mindfulness practice, positive re-evaluation training) [51]. Moreover, healthcare providers should pay attention to patients’ anxiety levels and create a supportive environment to help them reduce anxiety and improve sleep quality. Finally, given that resilience plays a moderating role on the relationship between rumination and anxiety symptoms, it may be a feasible measure to improve the level of resilience to solve sleep problems among patients with NAFLD. Interventions for resilience enhancement and stress management could be used to improve NAFLD patients’ resilience, such as positive psychology group intervention and mindfulness-based stress reduction [52].

Several limitations should be illustrated. First, this study was only carried out in one digestive disease treatment center in Northeast China. Thus, the findings should be cautiously generalized to other NAFLD populations across cultural backgrounds. Moreover, the exclusion of patients with a history of serious psychiatric illness from this study could cause difficulties in exactly interpreting the relationship between variables because the study mainly examined psychiatric variables (e.g., rumination, anxiety). Second, due to the cross-sectional design, the causal associations between rumination, anxiety symptoms, resilience, and sleep quality cannot be determined. Anxiety and sleep are linked through a self-reinforcing feedback loop. It was postulated that anxiety symptoms could cause poor sleep quality in this study. Conversely, sleep problems can exacerbate anxiety. Additionally, conceptual distinct between rumination and anxiety symptoms could be enough to suggest that rumination causes anxiety symptoms in this study [53], it seems equally plausible to arrange the study variables in multiple other configurations. Third, the self-administered method was adopted to measure our study variables, and there may be a recall or reporting bias that could influence the association among the variables. This potential influence was minimized by the use of high reliability and validity scales, anonymity protection, voluntary participation, and survey data quality control. Fourth, the study measured subjective sleep quality using PSQI, and did not include an objective measure of sleep (e.g., polysomnography) or a prospective monitoring subjective measure (e.g., sleep diary). In fact, there may be differences between subjective and objective sleep quality measurements, which affects the validity of the research results. Polysomnography is usually only performed in clinics or hospitals by specialized professional, and interferes with the patients, thus affecting sleep and possibly causing measurement deviation. Sleep diary relies on participants’ sustained adherence, and there is no standardized diary for research or clinical purposes [54].

## Conclusions

Rumination (including brooding and reflection) could be positively related to poor sleep quality, and anxiety symptoms had a significant role in accounting for the relationship among patients with NAFLD. Resilience showed a moderating role that could attenuate the positive associations of brooding, reflection and rumination with anxiety symptoms, which were gradually reduced as resilience increased. Thus, resilience could decline the roles of anxiety symptoms in accounting for the impacts of brooding, reflection and rumination on poor sleep quality. The findings suggested that interventions aimed at alleviating rumination, reducing anxiety symptoms, and enhancing resilience might help NAFLD patients sleep better.

## Supplementary Information

**Additional file 1.** Demographic and clinical characteristics in relation to anxiety symptoms and PSQI score.

## Data Availability

The datasets used and/or analyzed during the current study are available from the corresponding author on reasonable request.

## Abbreviations

NAFLD: Non-alcoholic fatty liver disease
NASH: Non-alcoholic steatohepatitis
PSQI: Pittsburgh sleep quality index
RRS: Ruminative responses scale
GAD-7: Generalized anxiety disorder 7-item
RS-14: 14-Item resilience scale
CNY: Chinese Yuan
BMI: Body mass index
ALT: Alanine aminotransferase
AST: Aspartate aminotransferase
CI: Confidence interval
SD: Standard deviation
