# Supplementary material for: Impact of rumination on sleep quality among patients with non‑alcoholic fatty liver disease: a moderated mediation model of anxiety symptoms and resilience
Source: BMC Psychiatry. 2023 Feb 2;23:84. doi: 10.1186/s12888-023-04572-8 (PMC9893673; doi:10.1186/s12888-023-04572-8)
Supplement: Supplementary file 1 — Additional file 1. Demographic and clinical characteristics in relation to anxiety symptoms and PSQI score. [file 12888_2023_4572_MOESM1_ESM.docx]

**Additional Table** Demographic and clinical characteristics in relation to anxiety symptoms and PSQI score

| **Variables** | **Anxiety symptoms** | ***F*/*t*** | ***P*** | **PSQI** | ***F*/*t*** | ***P*** |
| --- | --- | --- | --- | --- | --- | --- |
|  | **Mean ± SD** |  |  | **Mean ± SD** |  |  |
| Demographic characteristics |  |  |  |  |  |  |
| Age group |  | 0.401 | 0.670 |  | 4.482 | 0.012 |
| 18–39 years | 3.65 ± 4.22 |  |  | 4.54 ± 2.87 |  |  |
| 40–59 years | 3.91 ± 4.33 |  |  | 5.68 ± 3.61 |  |  |
| ≥ 60 years | 4.43 ± 4.52 |  |  | 5.89 ± 3.52 |  |  |
| Gender |  | 1.767 | 0.078 |  | 1.978 | 0.049 |
| Male | 3.42 ± 4.13 |  |  | 4.84 ± 3.31 |  |  |
| Female | 4.31 ± 4.43 |  |  | 5.62 ± 3.34 |  |  |
| Marital status |  | 0.899 | 0.369 |  | 0.273 | 0.785 |
| Married/cohabited | 3.75 ± 4.29 |  |  | 5.24 ± 3.26 |  |  |
| Single/divorced/widowed/separated | 4.38 ± 4.36 |  |  | 5.09 ± 3.78 |  |  |
| Educational level |  | 1.079 | 0.358 |  | 1.579 | 0.195 |
| Junior high school or below | 4.59 ± 5.16 |  |  | 5.88 ± 3.44 |  |  |
| Senior high school | 4.09 ± 4.29 |  |  | 5.51 ± 3.87 |  |  |
| Junior college | 3.32 ± 3.57 |  |  | 4.94 ± 3.02 |  |  |
| College or above | 3.60 ± 4.18 |  |  | 4.80 ± 3.07 |  |  |
| Place of residence |  | 1.616 | 0.113 |  | 0.073 | 0.942 |
| Urban | 3.62 ± 3.84 |  |  | 5.21 ± 3.33 |  |  |
| Rural | 5.28 ± 6.30 |  |  | 5.25 ± 3.45 |  |  |
| Occupation |  | 0.747 | 0.456 |  | 1.911 | 0.057 |
| Yes | 3.95 ± 4.42 |  |  | 5.01 ± 3.15 |  |  |
| No | 3.49 ± 3.85 |  |  | 5.92 ± 3.87 |  |  |
| Monthly household income |  | 3.482 | 0.032 |  | 3.770 | 0.024 |
| < 5,000 CNY | 4.56 ± 4.88 |  |  | 5.85 ± 3.67 |  |  |
| 5,000–10,000 CNY | 3.58 ± 3.98 |  |  | 4.78 ± 3.13 |  |  |
| > 10,000 CNY | 2.78 ± 3.13 |  |  | 4.70 ± 2.74 |  |  |
| Clinical characteristics |  |  |  |  |  |  |
| BMI group |  | 0.131 | 0.877 |  | 1.376 | 0.254 |
| < 24 kg/m^2^ | 3.81 ± 4.56 |  |  | 5.04 ± 3.41 |  |  |
| 24–28 kg/m^2^ | 3.98 ± 4.40 |  |  | 5.55 ± 3.61 |  |  |
| ≥ 28kg/m^2^ | 3.69 ± 4.05 |  |  | 4.84 ± 2.88 |  |  |
| Duration of NAFLD |  | 1.708 | 0.090 |  | 1.726 | 0.085 |
| ≤ 6 months | 3.54 ± 4.07 |  |  | 4.99 ± 3.20 |  |  |
| > 6 months | 4.53 ± 4.71 |  |  | 5.72 ± 3.61 |  |  |
| Disease severity |  | 2.757 | 0.008 |  | 2.888 | 0.004 |
| Simple fatty liver | 3.51 ± 4.07 |  |  | 4.98 ± 3.17 |  |  |
| Others | 5.79 ± 5.06 |  |  | 6.57 ± 3.98 |  |  |
| Comorbidities |  | 0.191 | 0.849 |  | 0.969 | 0.333 |
| Yes | 3.8 ± 4.15 |  |  | 5.39 ± 3.54 |  |  |
| No | 3.9 ± 4.48 |  |  | 5.01 ± 3.10 |  |  |
| Treatment |  | 0.326 | 0.745 |  | 0.397 | 0.691 |
| Yes | 3.95 ± 4.33 |  |  | 5.12 ± 3.37 |  |  |
| No | 3.78 ± 4.28 |  |  | 5.28 ± 3.33 |  |  |

BMI: body mass index; CNY: Chinese Yuan; NAFLD: non-alcoholic fatty liver disease; PSQI: Pittsburgh Sleep Quality Index; SD: standard deviation
